# Novel liquid biopsy based on comprehensive small RNA profiling for screening of cancer in dogs utilizing machine learning

**DOI:** 10.1093/jvimsj/aalag270

**Published:** 2026-10-30

**Authors:** Sora Harako, Masashi Yuki, Shunsuke Noguchi, Taisuke Ohno, Nobuhiro Nozaki, Ruisa Nishida, Hiroaki Hojo, Ririka Horikawa, Daiki Kawata, Yuichiro Noda, Naoki Miura, Takuya Mizuno, Yasuyuki Momoi

**Affiliations:** Research and Development Division, ARKRAY, Inc., Kyoto, Japan; Yuki Animal Hospital, Nagoya, Aichi, Japan; Department of Oncology, Japan Animal Referral Medical Center Osaka, Osaka, Japan; Department of Veterinary Clinical Pathobiology, Graduate School of Agricultural and Life Sciences, The University of Tokyo, Tokyo, Japan; Joint Graduate School of Veterinary Medicine, Kagoshima University, Kagoshima, Japan; Research and Development Division, ARKRAY, Inc., Kyoto, Japan; Research and Development Division, ARKRAY, Inc., Kyoto, Japan; Research and Development Division, ARKRAY, Inc., Kyoto, Japan; Research and Development Division, ARKRAY, Inc., Kyoto, Japan; Research and Development Division, ARKRAY, Inc., Kyoto, Japan; Joint Graduate School of Veterinary Medicine, Kagoshima University, Kagoshima, Japan; Veterinary Teaching Hospital, Joint Faculty of Veterinary Medicine, Kagoshima University, Kagoshima, Japan; Laboratory of Molecular Diagnostics and Therapeutics, Joint Faculty of Veterinary Medicine, Yamaguchi University, Yamaguchi, Japan; Division of Translational Research for One Medicine, Research Institute for Cell Design Medical Science, Yamaguchi University, Yamaguchi, Japan; Japan Small Animal Cancer Center, Affiliated with the Japan Small Animal Medical Center Foundation, Saitama, Japan; Department of Veterinary Clinical Pathobiology, Graduate School of Agricultural and Life Sciences, The University of Tokyo, Tokyo, Japan

**Keywords:** dog, machine learning, next-generation sequencing, noncoding RNA, oncology

## Abstract

**Background:**

Recent investigations have demonstrated the relevance in cancer of a variety of small RNAs, beyond microRNAs (miRNAs). However, the comprehensive application of these RNAs to cancer detection in dogs remains unexplored.

**Hypothesis/Objectives:**

We investigated whether combining miRNAs and other small RNAs using a custom database improves the accuracy of machine learning cancer screening in dogs compared with models using only miRNAs.

**Animals:**

A total of 389 dogs were enrolled: 85 with cancer, 77 with nonmalignant diseases, and 227 healthy controls.

**Methods:**

Small RNAs in blood samples were sequenced, and miRNAs (via miRBase) and other small RNAs (via a custom database) were profiled. Based on these profiles, 2 datasets were prepared: one with only miRNAs and another combining miRNAs with other small RNAs. For each dataset, 12 classification models were constructed using identical methods to enable paired comparisons of performance, with 5-fold cross-validation and area under the receiver operating characteristic curve (AUC) as the primary metrics.

**Results:**

The model that integrated both miRNAs and other small RNAs yielded a significantly higher AUC compared with the miRNA-only model (median AUC: 0.926 [95% CI, 0.911-0.944] vs 0.881 [0.860-0.897]; Wilcoxon signed-rank test, *P* < .001), indicating enhanced capability in discriminating malignant tumors in dogs.

**Conclusions and clinical importance:**

We investigated the comprehensive integration of diverse small RNA species, beyond miRNAs, using a machine learning approach for cancer screening in dogs. Although further prospective validation in independent cohorts is needed, our results indicate that this strategy improves the performance of cancer classification models in dogs.

## Introduction

Liquid biopsy has garnered increasing attention in recent years as a novel, minimally invasive method for early cancer detection.^1–3^ Cancer is a leading cause of mortality in dogs, and early diagnosis might broaden therapeutic options and improve survival rates.^4^^,^^5^ Because liquid biopsy allows for repeated testing, it is an ideal and promising tool for health checks and screening in veterinary medicine. Many dogs brought to animal hospitals present with a range of both neoplastic and non-neoplastic conditions.^6^^,^^7^ Practical cancer screening thus requires that dogs with cancer must be distinguishable not only from healthy dogs but also from those with nonmalignant diseases.

A number of new molecular markers are being explored for their potential to improve the accuracy of cancer detection. Sequencing methods for RNA enable the comprehensive profiling of a variety of small RNAs, including microRNAs (miRNAs). Studies examining classification models combining miRNA expression profiles with machine learning tools are beginning to yield useful information.^8–10^ We recently reported a miRNA-based machine learning model that distinguishes dogs with cancer from both healthy dogs and dogs with nonmalignant diseases.^9^ In addition to miRNAs, however, several other types of small RNAs have been reported as useful biomarkers in humans. These include small nucleolar RNAs, small nuclear RNAs (snRNAs), transfer RNA–derived fragments (tRFs), P-element induced wimpy testis–interacting RNAs (piRNAs), Y RNA, and ribosomal RNA–derived fragments (rRFs).^11–16^ The potential usefulness of these non-miRNA small RNAs as biomarkers in dogs is being increasingly recognized.^17–19^

In this context, we focused on the comprehensive utilization of small RNAs beyond miRNAs. However, the extent of the repertoire of small RNAs in dogs remains unclear because many small RNAs are still unregistered in existing databases. Therefore, we constructed a custom database that comprehensively covers sequences of small RNAs beyond miRNAs and used this database to evaluate the impact of integrating various small RNAs into cancer classification models.

## Materials and methods

### Study animals and ethics

In our retrospective cohort study, blood samples were collected from 389 dogs at multiple facilities in Japan between February and October 2024. The cancer group (*n* = 85) consisted of dogs diagnosed with cancer based on histopathology or cytology. The nonmalignant disease group (*n* = 77) included dogs diagnosed with nonmalignant conditions, and the healthy control group (*n* = 227) comprised dogs without abnormalities on medical examination and follow-up (Appendix, “Study animals and ethics”). Diagnostic criteria for group assignment were based on previous reports.^9^ All dogs were client-owned, and informed consent was obtained from the owners. Only surplus clinically collected specimens were analyzed.

### Sample collection and RNA extraction

Blood sample collection and RNA extraction were performed as previously described^9^ (Appendix, “Sample collection and RNA extraction”).

### Next-generation sequencing (NGS) library preparation and sequencing

The NGS library preparation, quantification, and sequencing were performed as previously described,^9^ with samples processed in duplicate (see Appendix, “NGS library preparation and sequencing”).

### Custom database construction

To expand the coverage of small RNA species, a custom database of reference sequences targeting non-miRNA small RNAs was constructed (Figure 1). The reference sequences were classified into 714 clusters, termed “other small RNA species” in our study (Appendix, “Custom database construction”).

**Figure 1:**
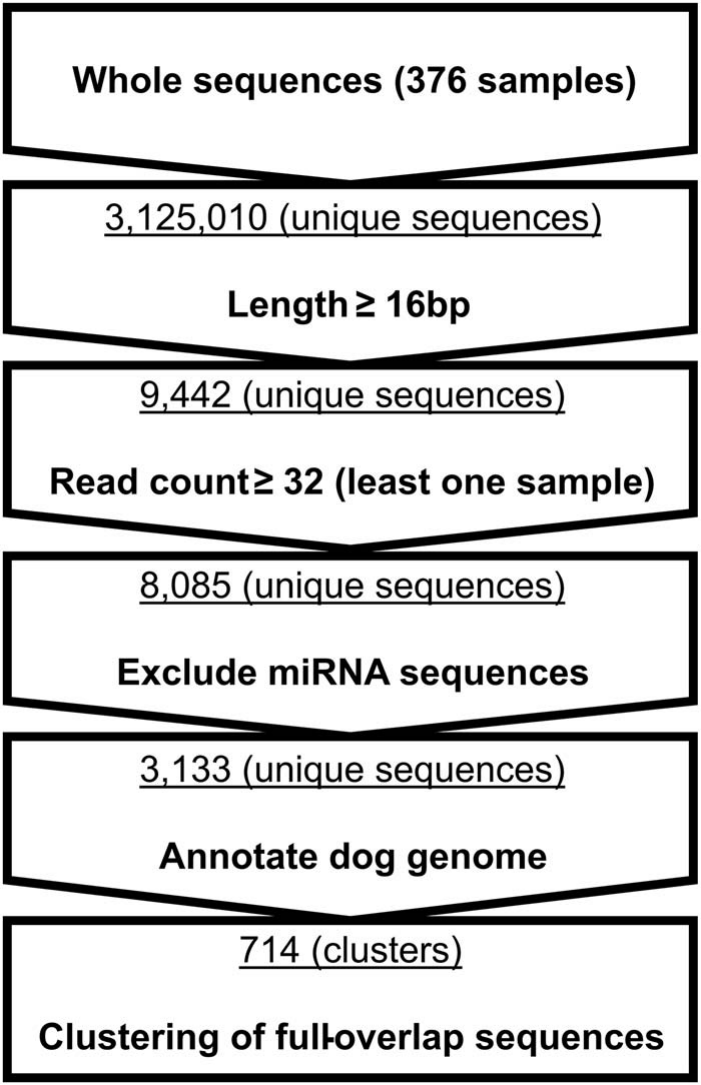
Overview of custom database construction from small RNA-seq data. Sequences were extracted from small RNA-seq data after adapter removal, filtering according to sequence length and read count, miRNA filtering, and annotation to the canine genome. Full-overlap sequence clustering then was performed to construct a custom database, which subsequently was used to develop a cancer classification model using machine learning. The 376 samples utilized for database construction were separate from those used for model development and evaluation. Abbreviation: miRNA = microRNA.

### Dataset preparation

Sequencing reads were separated into sample-specific FASTQ files based on index sequences. Adapter trimming, annotation using miRBase (miRNAs) and a custom database (other small RNAs), reads per million (RPM) normalization, and log2 transformation were then performed. As a result, 2 datasets were prepared: an “miRNA dataset” including only miRNAs, and a “combined dataset” integrating both miRNAs and other small RNA species (see Appendix, “Dataset preparation”).

### Cancer classification models

Cancer classification models were constructed using both the miRNA and combined datasets. For each dataset, 12 models were built by combining 4 feature selection methods with 3 classification algorithms, enabling paired comparisons under identical conditions (Figure 2). Model performance (the primary outcome) was evaluated as the median area under the receiver operating characteristic curve (AUC) from 5-fold cross-validation (see Appendix, “Cancer classification models”).

**Figure 2:**
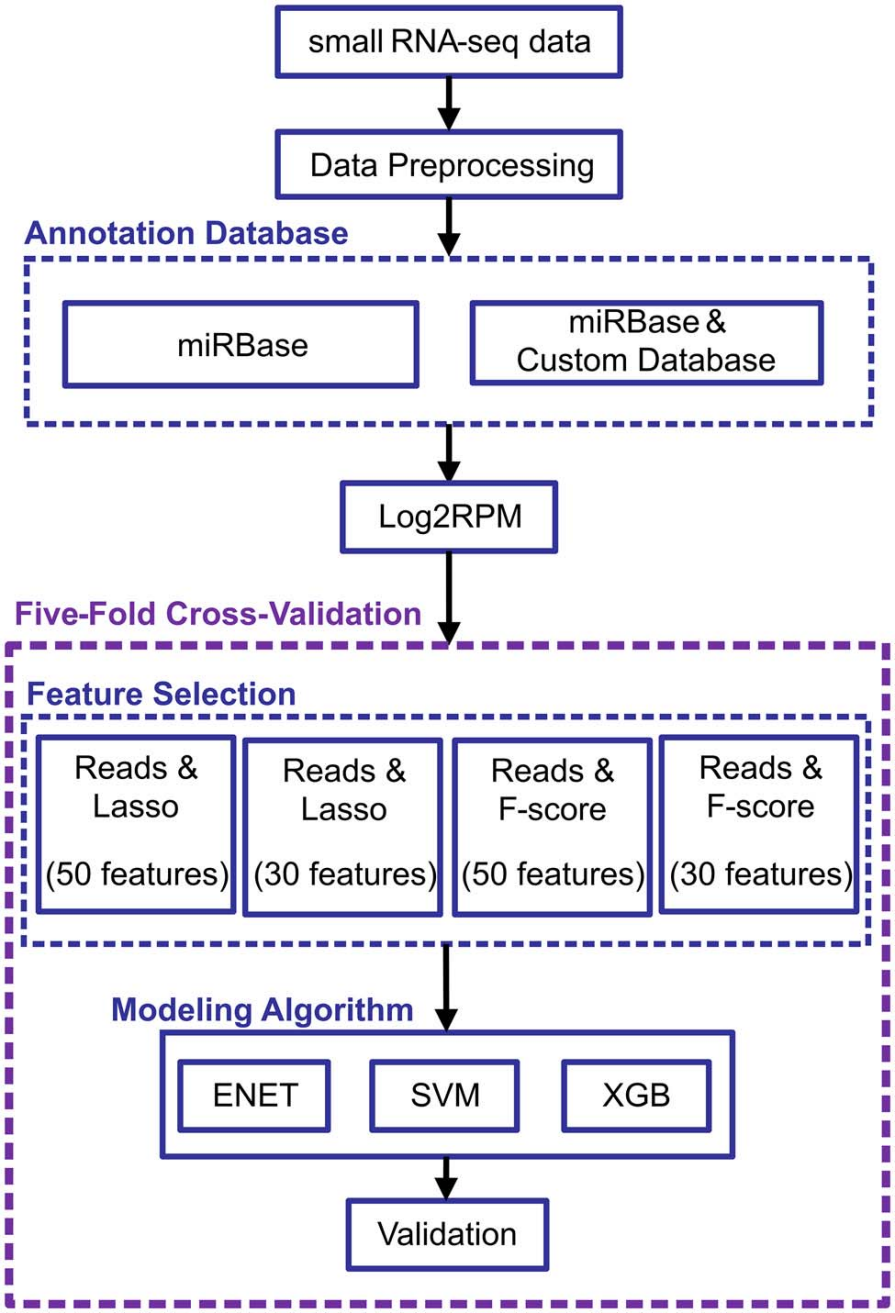
Process of dataset preparation, model development, and validation. Small RNA-seq data were annotated using 2 different methods after demultiplexing and adapter removal. Log_2_ RPM values calculated from these annotations were used as separate datasets in the subsequent model development and validation processes. Five-fold cross-validation was performed using all samples. In each cycle, 4 feature selection types were applied, followed by model construction using 3 different classifiers. Model performance was evaluated based on AUC values. Abbreviations: AUC = area under the receiver operating characteristic curve; ENET = elastic net regularization; F-score = Fisher discriminant ratio; LASSO = least absolute shrinkage and selection operator; RPM = reads per million; SVM = support vector machine with RBF kernel; XGB = eXtreme gradient boosting.

### Statistical analyses

Statistical analyses, including principal component analysis, correlation analysis, AUC calculation, and group comparisons, were conducted using R software (version 4.1.1). Median AUCs from the miRNA-only and combined datasets were paired by feature selection method and classifier, and compared using Wilcoxon signed-rank test, which does not assume normality of within-pair differences (see Appendix, “Statistical analyses”).

## Results

### Animal characteristics

The study included 389 dogs: 227 healthy controls, 77 with nonmalignant diseases, and 85 with cancer (Table 1). The median age of the healthy control, nonmalignant disease, and cancer groups was 87, 134, and 150 months, respectively. A significant age difference was observed between the cancer group and the other groups (*P* < .001, Welch’s *t-*test). The cohort included 58 intact males, 156 neutered males, 33 intact females, and 142 spayed females, with no significant differences in sex distribution among groups (*P* = .24, χ^2^ test). The most common breeds included in the study were mixed (*n* = 69), toy poodle (*n* = 63), miniature dachshund (*n* = 46), and chihuahua (*n* = 42). Dogs in the cancer group had a variety of cancer types (Table 2), and the nonmalignant disease group encompassed a range of conditions (Table 3). Although a significant age difference existed between groups, principal component analysis of the log_2_ RPM values of the top 50 miRNAs and other small RNA species identified no distinct age-based clusters, suggesting that the expression profiles of small RNAs were not strongly affected by age (Figure S1). Analyses of correlations between age and the expression levels of individual features further supported this observation (Figure S2). Here, “other small RNA species” refers to 714 clusters in the custom database (see “Custom database construction” in the Materials and methods section for details).

**Table 1:** Characteristics of dogs included in the study. Data regarding age, sex, and breed for all dogs included in the study: 227 healthy controls, 77 with nonmalignant diseases, and 85 with cancer. Median age and sex distribution for each group are shown. A significant age difference was observed between the cancer group and the other groups (*P* < .001, Welch’s *t-*test), whereas no significant differences were observed in terms of sex distribution (*P* = .24, χ^2^ test).

		**Healthy (*n* = 227)**	**Nonmalignant disease (*n* = 77)**	**Cancer (*n* = 85)**
**Age**	Median [months]	87	134	150
	Range [months]	6-200	28-219	19-207
**Sex**	Male	121 [53.3%]	41 [53.2%]	52 [61.2%]
	Intact	31	11	16
	Neutered	90	30	36
	Female	106 [46.7%]	36 [46.8%]	33 [38.8%]
	Intact	24	6	3
	Spayed	82	30	30
**Breed**	Mixed breed	49	7	13
	Toy Poodle	36	12	15
	Miniature Dachshund	20	14	12
	Chihuahua	26	11	5
	Shiba	19	4	2
	Miniature Schnauzer	11	3	7
	Yorkshire Terrier	11	4	1
	Pomeranian	8	3	3
	Maltese	7	0	0
	Other	40	19	27

**Table 2:** Cancer types. Dogs in the cancer group were affected by a variety of cancer types. The cancers listed in this table were classified primarily according to the cancer location classification provided by the Veterinary Society of Surgical Oncology (VSSO; https://vsso.org/dog-cancer-location).

**Location of tumor**	Number of samples	**Diagnosis**
**Oral**	18	Oral melanoma (17), oral squamous cell carcinoma (1)
**Skin**	12	Cutaneous mast cell tumor (12)
**Nasal Cavity**	10	Nasal cavity transitional cell carcinoma (1), nasal cavity chondrosarcoma (2), nasal sarcoma (1), nasal adenocarcinoma (5), nasal cavity squamous cell carcinoma (1)
**Testicles**	4	Seminoma (4)
**Bone**	3	Bone hemangiosarcoma (1), osteosarcoma (2)
**Mammary gland**	3	Inflammatory mammary carcinoma (2), malignant mixed mammary tumor (1)
**Liver**	3	Hepatocellular carcinoma (3)
**Anal gland**	3	Apocrine gland anal sac adenocarcinoma (3)
**Pancreas**	2	Insulinoma (2)
**Thyroid**	2	Thyroid carcinoma (2)
**Bladder**	2	Bladder transitional cell carcinoma (2)
**Mediastinum**	2	Thymic carcinoma (1), chemodectoma (1)
**Spleen**	1	Spleen hemangiosarcoma (1)
**Heart**	1	Cardiac hemangiosarcoma (1)
**Kidney**	1	Renal cell carcinoma (1)
**Colon**	1	Colon adenocarcinoma (1)
**Perianal**	1	Perianal adenocarcinoma (1)
**Others**	16	Chronic lymphocytic leukemia (1), multiple myeloma (2), soft tissue sarcoma (3), visceral sarcoma (3), multicentric lymphoma (2), gastrointestinal stromal tumor (1), malignant epithelial tumor (1), hemangiosarcoma of unknown primary (3)

**Table 3:** Types of nonmalignant diseases among dogs included in the study. Dogs in the nonmalignant disease group were affected by a variety of conditions, such as skin and subcutaneous diseases, hepatobiliary diseases, and others.

**Nonmalignant disease classification**	Number of samples
**Skin and subcutaneous diseases**	20
**Hepatobiliary diseases**	9
**Diseases of the nervous system**	9
**Cardiovascular diseases**	7
**Urological diseases**	6
**Gastrointestinal diseases**	5
**Ocular diseases**	5
**Endocrine diseases**	4
**Respiratory diseases**	3
**Immune-mediated diseases**	2
**Orthopedic diseases**	1
**Other diseases**	6

### Reproducibility of measurements

Library preparation encompassed 16 separate batches, with control samples derived from the same pooled plasma (provided by Oriental Bio Inc., Yamaguchi, Japan) included in each batch. Analysis of the correlation in log_2_ RPM for the top 50 miRNAs and other small RNA species between control samples across batches showed *R*^2^ ≥ .97 in all comparisons, indicating high measurement stability (Figure 3).

**Figure 3:**
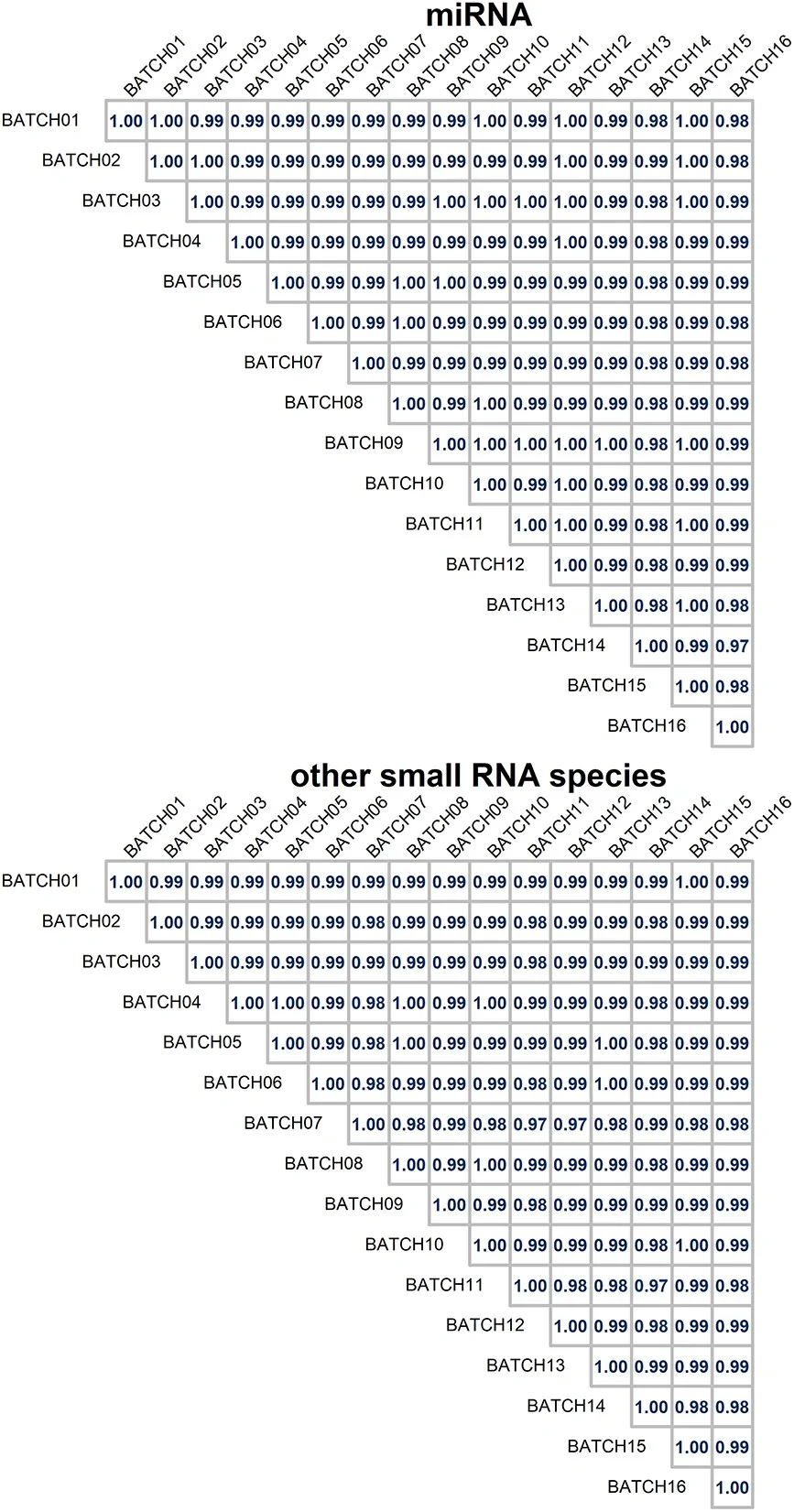
Pairwise correlation analysis of the top 50 features. RNA derived from the same pooled plasma was used as a control in all 16 library preparation batches, and correlations of log_2_ RPM values were compared among the top 50 miRNAs and other small RNA species. *R*^2^ values were ≥ .97 for all comparisons, indicating a high degree of measurement stability. Abbreviations: miRNA = microRNA; RPM = reads per million.

### Expression levels of miRNAs and other small RNA species

The miRNAs and other small RNA species with ≥ 20 reads in at least 90% of samples were selected from the full dataset (Figure S3), resulting in 88 consistently detected (ie, stable) miRNAs and 39 other small RNA species.

### Cancer classification models

For each cross-validation fold, 12 classification models were constructed using both the miRNA and combined datasets. The term “combined dataset” here refers to an integrated dataset comprising both miRNAs and other small RNA species (see “Dataset preparation” in the Materials and methods section for details). Figure 4 and Table S1 show the median AUC values from 5-fold cross-validation, stratified by feature selection method and modeling algorithm. Models developed using the combined dataset consistently exhibited higher AUC values than models developed using only miRNA data, regardless of the feature selection method or modeling algorithm applied. The median AUC for the combined dataset was 0.926 (95% CI, 0.911-0.944), whereas the median AUC for the miRNA-only dataset was 0.881 (95% CI, 0.860-0.897). This improvement was statistically significant (Wilcoxon’s signed-rank test, *P* < .001). Building on this finding, we further evaluated model sensitivity from a clinical perspective at a fixed specificity of 0.90. We also conducted exploratory subgroup analyses for major types of cancer encountered in clinical practice,^20–22^ given the heterogeneity of cancer types. Because the model development cohort included only 2 lymphoma cases, additional lymphoma cases collected during a different period were evaluated as a separate validation cohort (Table S2). Although sensitivity across cancer types varied among models, the combined dataset showed significant improvement in sensitivity over the miRNA-only dataset (Figure S4).

**Figure 4:**
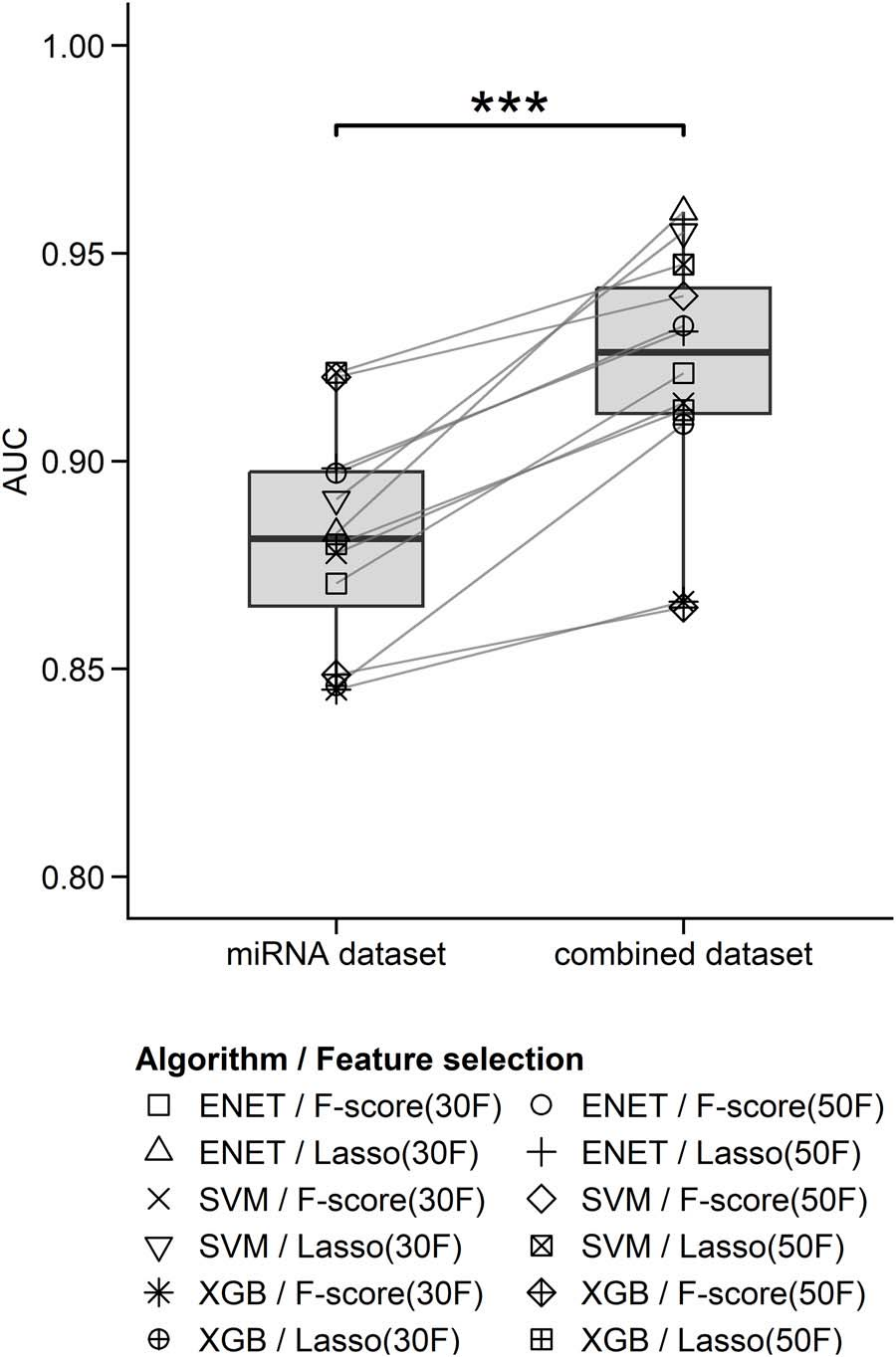
Comparison of the distribution of AUC values between datasets. The figure shows the distribution of median AUC values from 5-fold cross-validation of cancer classification models constructed using each of the miRNA and combined datasets. Methods using the same feature selection and algorithm are connected by solid lines. A significant increase in AUC values was observed with the combined dataset (Wilcoxon’s signed-rank test; ^***^P < .001). The median AUC for the miRNA-only dataset was 0.881 (95% CI, 0.860-0.897), and the median AUC for the combined dataset was 0.926 (95% CI, 0.911-0.944). Abbreviations: AUC = area under the receiver operating characteristic curve; miRNA = microRNA.

### Comparative differential expression profiles of several small RNA species

To determine why use of the combined dataset improved discrimination, we compared the log_2_ RPM distributions of selected features between the cancer and healthy control and nonmalignant disease groups. For the clusters of other small RNA species used in the representative model shown in Figure 4, representative sequences were identified using RNAcentral, and Table 4 summarizes those with ≥ 95% coverage and 100% identity (https://www.rnacentral.org/).^23^ Notably, several clusters, identified as ribosomal RNA, Y RNA, and snRNA, showed significant quantitative differences among the groups (Figure 5), which we speculate contributed to the improved discriminatory performance.

**Table 4:** RNA types of cluster representative sequences. Representative sequences from clusters used as features in the model shown in Figure 4 were queried against RNAcentral. Table shows some of the sequences exhibiting ≥ 95% coverage and 100% identity.

**Cluster**	**Representative sequence**	**RNAcentral identifier**	**RNA type**
**Cluster-4**	CGACTCTTAGCGGTGGATCACTCGGCTCGTGCGTCGATGAAGAACGCAGC	URS000080E170_9615	rRNA
**Cluster-5**	GTACTTGGATGGGAGACCGCCTGGGAATACCGGGTGCTGTAGGCTTT	URS000062DC62_9615	
**Cluster-82**	GCATGAGCGCACGTGTTAGGACCCGAAAGATGGTGAAC	URS00022AF078_9615	
**Cluster-101**	AAGAACGAAAGTCGGAGGTTCGAAGACGATCAGATAC	URS000063759B_9615	
**Cluster-26**	ACGGCCCTGGCGGAGTGCTGAGAAGACGGTCGAACTTGACT	URS000063759B_9615	
**Cluster-6**	AACACGGGAAACCTCACCCGGCCCGGACACGGACAGGATTGACAGAT	URS000063759B_9615	
**Cluster-46**	ATTGGAGGAGTAAGAGGGAGAGAACGCAGTCTGAGTGGT	URS000062935D_9615	snoRNA
**Cluster-1**	TCGACTGCATAATTTGTGGTAGTGGGGGACTGCGTTCGCGCTTTCCCCTG	URS000032B6B6_9615	snRNA
**Cluster-106**	TTGCACTCCGGATGTGCTGACCCCTGCGATTTCCCC	URS000032B6B6_9615	
**Cluster-17**	TTTGTTCCTTCTCCACTCCCACTGCTTCACTTGACCGGCCTT	URS0000689324_9615	Y RNA
**Cluster-267**	GGCTGGTCCGAGTGCAGTGGTGCTTACAATT	URS0000689324_9615	
**Cluster-31**	GGCTGGTCCGATGGTAGTGGGTTATCAGAACTTATTAACA	URS00006B9ABD_9615	
**Cluster-59**	TCCCTGGTGGTCTAGTGGTTAGGATTCGGCGCTCTCACC	URS00004F0321_9615	tRNA

**Figure 5:**
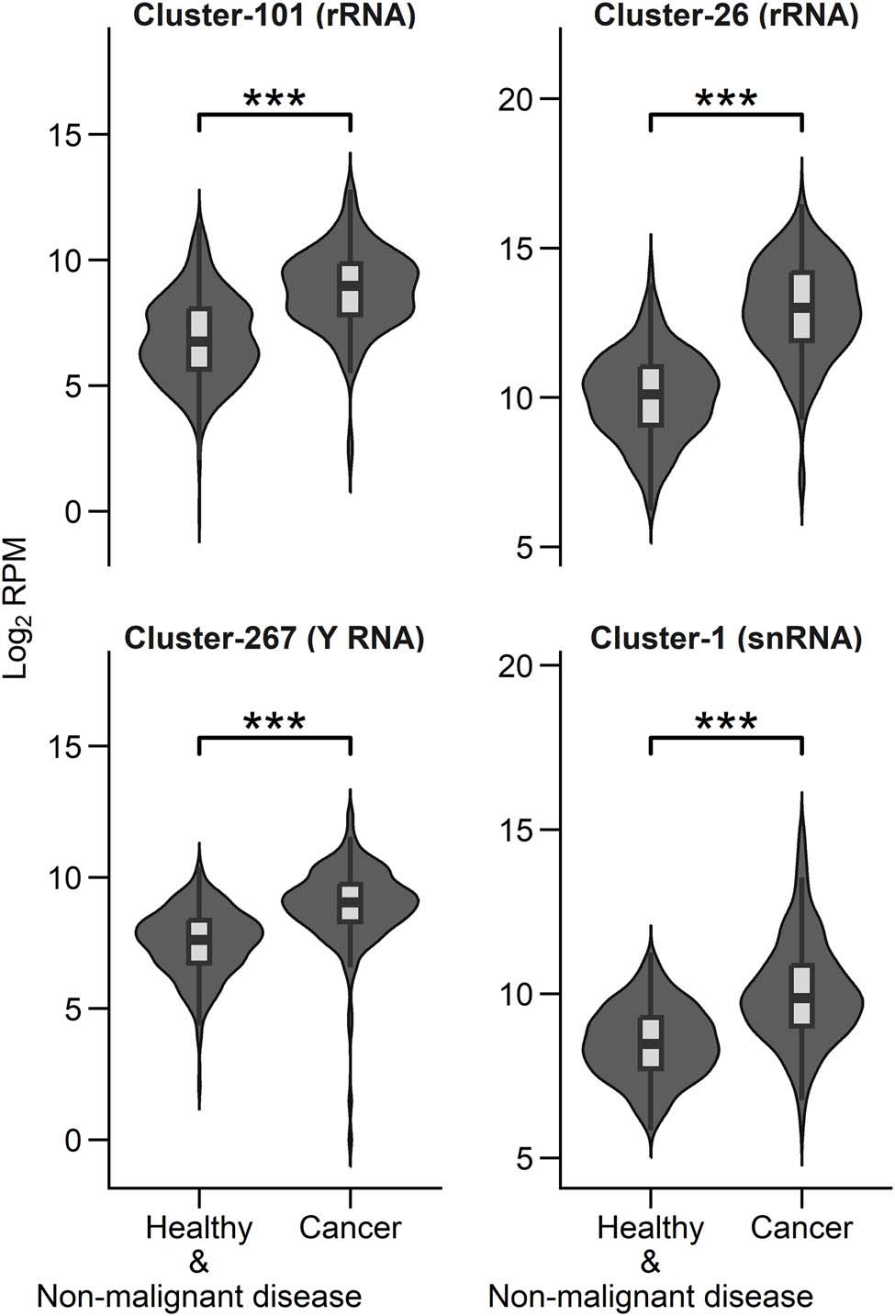
Log_2_ RPM distributions in the cancer vs healthy control/nonmalignant disease groups. Among features in the combined dataset, some showed clear differences in log_2_ RPM values between the cancer group and the healthy control/nonmalignant disease groups (Wilcoxon rank-sum test with continuity correction). ***P < .001. Abbreviation: RPM = reads per million.

## Discussion

Our study comprehensively analyzed small RNA profiles in dogs and identified 3 key findings. First, integrating several other small RNA species, such as rRFs, snRNAs, and Y RNAs, in addition to known miRNAs, significantly improves the performance of cancer discrimination models (as measured by AUC). Second, small RNA species other than miRNAs are stable and can be consistently detected in plasma. Third, in addition to Y RNAs and snRNAs, rRFs might serve as useful biomarkers for discriminating cancer in dogs.

Our results showed that combined analysis of miRNAs and other small RNAs increases the AUC of cancer discrimination models used in dogs. This enhanced performance was consistent regardless of the machine learning algorithm or feature selection method used. Consistent with these results, the sensitivity for cancer detection at a fixed specificity of 0.90 was also significantly enhanced after incorporation of additional small RNA species into the model (Figure S4). Although individual small RNAs, such as Y RNAs and snRNAs, have been identified as useful cancer biomarkers in dogs, previous research has largely been limited to evaluating these RNAs in isolation as single biomarker candidates.^18^^,^^19^ Our study demonstrated the diagnostic advantage of incorporating a broad spectrum of small RNA profiles into a machine learning–based model for cancer discrimination in dogs.

We also found that some other small RNA species included in our custom database are stably expressed and thus consistently measurable in plasma in amounts comparable to miRNAs. These non-miRNA small RNA species exhibited high coefficients of determination between assay runs (*R*^2^ = 0.97-1.00), and 39 species met the criterion of ≥ 20 reads in > 90% of all samples. Previous studies have focused primarily on the abundance and stability of circulating miRNAs. Our results, however, indicate that some non-miRNA small RNAs also can be reproducibly and reliably measured in plasma, thereby expanding the potential pool of biomarkers suitable for use in cancer screening.^24^^,^^25^

The classification models selected multiple rRFs as important features, suggesting that rRFs should be considered candidate diagnostic markers for identifying cancer in dogs. Of the 13 features identified using RNAcentral, 6 were rRFs, 3 were Y RNAs, and 2 were snRNAs. The Y RNAs and snRNAs have been linked to cell proliferation and aberrant RNA splicing, respectively, suggesting their potential involvement in tumor-related biological processes.^26^^,^^27^ Several rRFs also have been associated with Argonaute proteins, suggesting their potential involvement in cancer-related post-transcriptional gene regulation.^28^^,^^29^ Furthermore, altered plasma rRF concentrations might, at least in part, reflect dysregulation of ribosome biogenesis and rRNA processing in tumor cells.^30^

Nevertheless, rRFs remain one of the least studied small RNA species in cancer classification research. Several studies in humans identified rRFs as potential predictive markers for the early recurrence of prostate cancer, and these rRFs are also reportedly present in abundance in extracellular vesicles associated with several cancer cell lines.^31^^,^^32^ Altered expression of rRFs also has been observed in exosomes derived from cancer cell lines in dogs, and our findings are consistent with these previous reports.^18^ Together with our observations of the small RNA profiles in plasma, these findings highlight the potential usefulness of rRFs as cancer biomarkers. By extending the insights gained from previous research, our study emphasizes the importance of further exploring the diagnostic and biological relevance of rRFs in cancer.

The integrative small RNA model constructed in our study distinguished dogs with cancer from both healthy dogs and dogs with nonmalignant diseases with a high degree of accuracy, indicating its potential utility as a diagnostic tool in clinical settings. The model achieved consistent discriminative performance, even with a decreased number of features. This attribute of the model is particularly encouraging, because it could help decrease the likelihood of overfitting and enhance interpretability. Our results could contribute to the practical implementation of cancer screening and early diagnosis in dogs. Furthermore, the integrative use of novel small RNA species, including rRFs, indicates an even broader potential for multifaceted biomarker discovery than has been explored previously.

Our study had some limitations. First, because the custom database was designed primarily for comprehensiveness, detailed annotation against existing non-miRNA databases was not performed. In addition, we cannot completely exclude the possibility that some sequences in the custom database were derived from DNA rather than RNA. We also note that the library preparation method used in our study (adapter ligation) is susceptible to the effects of RNA terminal modifications and structure, thus potentially decreasing the efficiency of detecting certain small RNA species, such as piRNAs.^33^^,^^34^ In addition, we applied a read-based filtering step, regardless of expression direction, to exclude low-abundance features that are more susceptible to technical variability. Consequently, features markedly downregulated in cancer also might have been excluded. Addressing these issues lies beyond the scope of our study and will require further investigation. Second, because factors such as age, sex, and breed could not be perfectly matched between the positive and negative classes in our study, these variables could have confounded the results. However, each model generally indicated that these factors had minimal impact on prediction scores (Table S3). In one model, a significant difference among breeds was noted. Nonetheless, the distinction between the positive and negative classes within that breed remained clear, suggesting that the overall effect of these variables was limited. Although age was not strongly associated with prediction scores or expression levels, dogs in the cancer group were significantly older than dogs in the other groups, and residual confounding associated with age therefore cannot be completely excluded. Third, although the model was evaluated using 5-fold cross-validation, as recommended in a previous study,^35^ challenges related to overfitting and generalizability still remain in machine learning applications. In addition, although our study provides evidence that incorporating non-miRNA small RNAs can enhance diagnostic performance, the model development cohort included limited numbers of several malignant tumors common in dogs, including only 2 lymphoma cases. To address this limitation, we conducted an additional evaluation using a separate cohort of lymphoma cases collected during a different period and confirmed that sensitivity was maintained. Nevertheless, validation in larger and more representative cohorts reflecting real-world clinical prevalence remains essential to confirm the broader clinical generalizability of the added diagnostic value observed in our study. An additional limitation is that several tumor types were represented by only a few cases in the model development cohort. Validation in larger cohorts with adequate representation of individual tumor types therefore will be needed to determine whether model performance is driven primarily by molecular signatures shared across malignancies or by tumor-specific characteristics. Additional sample collection is ongoing to support future independent validation in larger and more representative cohorts. Finally, understanding of the biological functions of many non-miRNA species, particularly rRFs, remains limited, emphasizing the need for further fundamental research. Nonetheless, the utility of these small RNAs in classification models does not depend on a full understanding of their mechanistic roles, and their reproducible detection patterns across large sample sets support their potential as practical biomarkers, even in the absence of detailed functional characterization.

In conclusion, we determined that integrated analysis of non-miRNA small RNAs along with miRNAs can significantly improve the performance of cancer discrimination models in dogs. The novel contribution and measurement stability of small RNA species such as rRFs identified in our study indicate that the prospects for future biomarker development and clinical applications are promising. Prospective external validation in independent, clinically representative cohorts will be required before clinical implementation, and the resulting data are expected to further clarify and establish the clinical utility of this approach.

## Supplementary Material

Supplementary_material_aalag270

## Off-label antimicrobial declaration

The authors declare no off-label use of antimicrobials.

## Institutional animal care and use committee or other approval declaration

The authors declare no institutional animal care and use committee or other approval was required.

## Appendix: Supplementary methods

### Study animals and ethics

Canine blood samples were collected between February 2024 and October 2024 at Yuki Animal Hospital (Nagoya, Japan), the Animal Testing Center (Nagoya, Japan), and JARMeC (Minoh, Japan). The overall sample size was determined with the goal of exceeding that of previous studies,^8^ and specimens up to the maximum number possible were collected within the study period. The cohort comprised 389 dogs of various ages and differing health status. The cancer group (*n* = 85) consisted of dogs diagnosed with cancer via pathology or cytology, with plasma samples collected before therapeutic intervention at the initial visit. The nonmalignant disease group (*n* = 77) included dogs diagnosed with conditions other than cancer based on physical examinations by veterinarians, blood tests, histologic examinations, cytology, or thoracic radiography, which confirmed the absence of any tumors. Moreover, in accordance with our previously reported methods, these dogs underwent 3 months of follow-up, during which no tumor development was detected by these examinations. Plasma samples from this group were collected at initial, follow-up, or health check visits, with some obtained after therapeutic intervention. The healthy control group (*n* = 227) comprised dogs with no abnormalities detected in the above examinations and no abnormal findings at a 3-month follow-up. Blood samples for plasma preparation were collected from dogs in the healthy control group during routine health check-ups. All dogs were client-owned, and informed consent for all study procedures was obtained from the owners. All samples examined in the study were surplus portions of clinically necessary specimens collected from each dog and analyzed after clinical testing.

### Sample collection and RNA extraction

All blood samples were transferred into micro blood collection tubes (MiniCollect; Sekisui Medical, Tokyo, Japan) within 2 h of syringe collection, followed by centrifugation to separate plasma. The separated plasma then was transferred to microtubes and mixed with a nucleic acid stabilizer. Plasma samples showing hemolysis after centrifugation were excluded. Samples were stored at −80°C until RNA extraction. Total RNA, including miRNAs, was extracted from plasma using Maxwell RSC miRNA from Tissue or Plasma and Serum kits (Promega, Madison, WI, USA). During the extraction process, deoxyribonuclease treatment was performed according to the manufacturer’s instructions to remove contaminating genomic DNA. The RNA samples were stored at −80°C until preparation of next-generation sequencing (NGS) libraries.

### NGS library preparation and sequencing

Extracted RNA was used to generate a library for NGS analysis. An automated liquid-handling system was used for pipetting (Agilent Bravo NGS; Agilent Technologies Inc., Santa Clara, CA, USA). Library construction followed a previously published protocol.^36^ Libraries were quantified using a TapeStation HS D1000 system (Agilent Technologies), pooled to a uniform concentration, and sequenced using an Ion S5 system (Thermo Fisher Scientific, Waltham, MA, USA). Each RNA sample was processed twice for library construction and sequencing.

### Custom database construction

A custom database was constructed to maximize the utility of the small RNA sequencing data. Only minimal filtering was applied in order to ensure comprehensiveness and retain potentially informative features for machine learning–based cancer classification. As outlined in Figure 1, reference sequences were generated from all reads collected from 376 samples; the samples used for database construction were prepared separately from those used for model development and evaluation.

Cutadapt (ver. 2.8) was used to trim adapter sequences from reads in each sample, and unique sequences were extracted.^37^ Sequences shorter than 16 nucleotides and sequences with a total read count of < 32 across all samples were removed. Across all samples analyzed using this process, the mean total read count was 999 152, and the lowest read count observed was 336 380. Sequences not mapped to the canine miRNA reference (miRBase 22.1, via miraligner ver. 3.5) were further annotated against the dog genome (canFam4) using miRDeep2 (ver. 0.1.3), and the corresponding sequences were collected.^38–41^

The sequences resulting from the above process were clustered using CD-HIT (ver. 4.6.4), with a 100% identity threshold, forming clusters that were numbered 1-714.^42^ CD-HIT clusters sequences by first sorting them according to length, designating the longest sequence as the initial cluster representative, and then comparing each remaining sequence to the existing representatives. Clustering occurs only when a sequence is fully and contiguously contained within a representative sequence; otherwise, new clusters are formed. In each cluster, the longest sequence is selected as the representative. In contrast to miRNAs, these 714 clusters were collectively referred to in this study as “other small RNA species.”

### Dataset preparation

Sequencing reads were initially separated into FASTQ files for each sample according to index sequences. The resulting sample-specific files were then processed as follows: adapter sequences were removed using Cutadapt; annotation was performed against *Canis lupus familiaris* sequences in miRBase (v22.1) using miraligner and subsequently against the custom database using miRDeep2. For each miRNA or other small RNA species, the total number of reads mapped to that reference was determined as the read count. Read counts for both miRNAs and other small RNA species were normalized to reads per million (RPM) using the total miRNA read count as the denominator and log_2_-transformed using the following equation:

log_2_([read count of each miRNA or other small RNA species]/[total miRNA read count] × 10^6^ + 1).

This calculation generated relative expression levels for miRNAs (from miRBase) and other small RNA species (from the custom database). Two datasets were thus created: an “miRNA dataset” containing only miRNAs and a “combined dataset” integrating both miRNAs and other small RNA species.

### Cancer classification models

Both the miRNA and combined datasets were used to construct machine learning–based models for cancer classification. All samples were randomly divided into 5 folds for cross-validation, ensuring that replicate samples were placed within the same fold. In each cross-validation cycle, 4 folds were used for training, and 1 fold was used for validation. Feature selection was performed strictly within the training folds of each cross-validation cycle, so that no information from the validation fold was incorporated into the feature selection process, thereby avoiding potential data leakage. For each dataset, models were constructed using 4 feature-selection methods and 3 modeling algorithms, resulting in 12 different models for each dataset. Details regarding model construction and validation are provided in the section below. Figure 2 provides an overview of the workflow from small RNA-seq data output to model development and validation.

### Model building and validation

Feature selection was carried out in 2 stages using the training data. First, miRNAs with a read count ≥ 50 in at least 90% of all samples, and other small RNA species with a read count ≥ 20 in at least 90% of samples, were retained. Second, least absolute shrinkage and selection operator (LASSO) and the Fisher discriminant ratio (F-score) were applied to select the top approximately 50 and 30 features, respectively, yielding 4 feature sets. To account for the potential impact of the number of features on model performance, the same number of features was selected for each dataset.

For each feature set, classification models were constructed using 3 classifiers: Elastic Net Regularization (ENET), Support Vector Machine with RBF kernel (SVM), and eXtreme Gradient Boosting (XGB). These algorithms were intentionally selected due to their diversity, as they represent fundamentally different approaches: ENET for regularized linear classification, SVM for kernel-based nonlinear classification, and XGB for tree-based ensemble classification. Given the imbalance in class sizes across the cancer, healthy control, and nonmalignant disease groups, class weighting was applied during training for all classifiers to prevent model bias toward the majority classes.

Model performance was evaluated based on the area under the receiver operating characteristic curve (AUC) for the validation set. The R packages *glmnet* and *caret* were used for LASSO, F-score, ENET, SVM, and XGB analyses, whereas AUC was calculated using the *pROC* package.

### Statistical analyses

All statistical analyses were performed using R software, version 4.1.1 (R Core Team [2021]; R: A language and environment for statistical computing. R Foundation for Statistical Computing, Vienna, Austria, https://www.R-project.org/). Data were handled and visualized using dplyr, tidyr, and ggplot2. Principal component analysis was performed using the prcomp function. Correlation matrices were visualized using the corrplot package. The AUC values were calculated using the pROC package. The statistical significance of differences between groups was assessed using the Wilcoxon rank-sum test or Kruskal–Wallis test, as appropriate. Post hoc pairwise comparisons after significant Kruskal–Wallis test results were conducted using Dunn’s test with Bonferroni correction. Medians and 95% CIs were estimated via bootstrap resampling (5000 iterations, boot package).

## Abbreviations

AUC: area under the receiver operating characteristic curve
DNA: deoxyribonucleic acid
ENET: elastic net regularization
F-score: Fisher discriminant ratio
LASSO: least absolute shrinkage and selection operator
miRNA: microRNA
NGS: next-generation sequencing
piRNA: P-element induced wimpy testis–interacting RNAs
RNA: ribonucleic acid
rRF: ribosomal RNA–derived fragment
rRNA: ribosomal RNA
RPM: reads per million
SVM: support vector machine
snRNA: small nuclear RNA
snoRNA: small nucleolar RNA
tRF: transfer RNA–derived fragment
VSSO: Veterinary Society of Surgical Oncology
XGB: eXtreme gradient boosting

## References

[1] Ma L, Guo H, Zhao Y, et al. Liquid biopsy in cancer current: status, challenges and future prospects. Signal Transduct Target Ther. 2024;9:336. 10.1038/s41392-024-02021-w PMC11609310 39617822

[2] Liang X, Tang Q, Chen J, Wei Y. Liquid biopsy: a breakthrough technology in early cancer screening. Cancer Screen Prev. 2025;4:40-52. 10.14218/CSP.2024.00031

[3] Flory A, McLennan L, Peet B, et al. Cancer detection in clinical practice and using blood-based liquid biopsy: a retrospective audit of over 350 dogs. J Vet Intern Med. 2023;37:258-267. 10.1111/jvim.16616 PMC9889684 36661398

[4] Inoue M, Sugiura K. Dōbutsu byōin karute dēta o moto ni shita Nihon no inu to neko no jumyō to shibō gen'in bunseki [Analysis of life expectancy and causes of death of dogs and cats in Japan based on veterinary medical record data]. Nihon Juishikai Zasshi. 2022;75:e128-e133 (in Japanese). 10.12935/jvma.75.e128

[5] Fleming JM, Creevy KE, Promislow DE. Mortality in North American dogs from 1984 to 2004: an investigation into age-, size-, and breed-related causes of death. J Vet Intern Med. 2011;25:187-198. 10.1111/j.1939-1676.2011.0695.x 21352376

[6] Uchino T . Inu no shinsikkan no hassei jōkyō [Incidence of heart disease in dogs]. Jpn J Electrocardiology. 2002;22:40-44 (in Japanese). 10.5105/jse.22.Suppl1_40

[7] Uchino T . Shōdōbutsu rinshō dēta no shūshū oyobi ekigaku e no ōyō [Collection of clinical data in small animals and its application to epidemiology]. Juui Ekigaku Zasshi. 1999;2:73-76 (in Japanese). 10.2743/jve.3.73

[8] Omura K, Ide K, Takahashi M, et al. Development of a sensitive disease-screening model using comprehensive circulating microRNA profiles in dogs: a pilot study. Vet Anim Sci. 2025;27:100414. 10.1016/j.vas.2024.100414 PMC11647647 39691815

[9] Nishida R, Ide K, Takahashi M, et al. A machine learning model for cancer screening in dogs using comprehensive circulating microRNA profiles. J Vet Intern Med. 2026;40:aalaf071. 10.1093/jvimsj/aalaf071 PMC12859747 41742549

[10] Chokeshaiusaha K, Sananmuang T, Puthier D, Nguyen C. A novel cross-species differential tumor classification method based on exosome-derived microRNA biomarkers established by human-dog lymphoid and mammary tumor cell lines' transcription profiles. Vet World. 2022;15:1163-1170. 10.14202/vetworld.2022.1163-1170 PMC9210832 35765483

[11] Hu X, Cui W, Liu M, et al. SnoRNAs: the promising targets for anti-tumor therapy. J Pharm Anal. 2024;14:101064. 10.1016/j.jpha.2024.101064 PMC11613181 39634568

[12] Dong X, Ding S, Yu M, et al. Small nuclear RNAs (U1, U2, U5) in tumor-educated platelets are downregulated and act as promising biomarkers in lung cancer. Front Oncol. 2020;10:1627. 10.3389/fonc.2020.01627 PMC7434840 32903345

[13] Yu M, Lu B, Zhang J, Ding J, Liu P, Lu Y. tRNA-derived RNA fragments in cancer: current status and future perspectives. J Hematol Oncol. 2020;13:121. 10.1186/s13045-020-00955-6 PMC7487644 32887641

[14] Han YN, Li Y, Xia SQ, Zhang YY, Zheng JH, Li W. PIWI proteins and PIWI-interacting RNA: emerging roles in cancer. Cell Physiol Biochem. 2017;44:1-20. 10.1159/000484541 29130960

[15] Guglas K, Kolenda T, Stasiak M, et al. YRNAs: new insights and potential novel approach in head and neck squamous cell carcinoma. Cells. 2020;9:1281. 10.3390/cells9051281 PMC7290662 32455790

[16] Yang P, Li Z, Chen X, et al. Non-canonical small noncoding RNAs in the plasma extracellular vesicles as novel biomarkers in gastric cancer. J Hematol Oncol. 2025;18:39. 10.1186/s13045-025-01689-z PMC11966915 40176097

[17] Rahman MM, Lai YC, Husna AA, et al. Aberrantly expressed snoRNA, snRNA, piRNA and tRFs in canine melanoma. Vet Comp Oncol. 2020;18:353-361. 10.1111/vco.12558 31769925

[18] Husna AA, Rahman MM, Chen HW, Hasan MN, Nakagawa T, Miura N. Long non-coding RNA and transfer RNA-derived small fragments in exosomes are potential biomarkers for canine oral melanoma. Vet Comp Oncol. 2022;20:653-663. 10.1111/vco.12818 35388611

[19] Hasan MN, Rahman MM, Husna AA, Nozaki N, Yamato O, Miura N. YRNA and tRNA fragments can differentiate benign from malignant canine mammary gland tumors. Biochem Biophys Res Commun. 2024;691:149336. 10.1016/j.bbrc.2023.149336 38039834

[20] Irie M . Kokunai ichiji shinryō dōbutsu byōin 26 shisetsu ni okeru inu to neko no shuyō hassei jōkyō chōsa [Survey on tumor incidence in dogs and cats at 26 primary veterinary clinics in Japan]. Nihon Juui Ishikai Zasshi. 2016;69:468-473. (in Japanese). 10.12935/jvma.69.468

[21] He X, Gao Y, Deng Y, et al. The comparative oncology of canine malignant melanoma in targeted therapy: a systematic review of in vitro experiments and animal model reports. Int J Mol Sci. 2024;25:10387. 10.3390/ijms251910387 PMC11476434 39408717

[22] O'Keefe DA . Canine mast cell tumors. Vet Clin North Am Small Anim Pract. 1990;20:1105-1115. 10.1016/S0195-5616(90)50087-X 2115219

[23] Petrov AI, Kay SJE, Gibson R, et al. RNAcentral: an international database of ncRNA sequences. Nucleic Acids Res. 2015;43:D123-D129. 10.1093/nar/gku991 PMC4384043 25352543

[24] Chen X, Ba Y, Ma L, et al. Characterization of microRNAs in serum: a novel class of biomarkers for diagnosis of cancer and other diseases. Cell Res. 2008;18:997-1006. 10.1038/cr.2008.282 18766170

[25] Mitchell PS, Parkin RK, Kroh EM, et al. Circulating microRNAs as stable blood-based markers for cancer detection. Proc Natl Acad Sci U S A. 2008;105:10513-10518. 10.1073/pnas.0804549105 PMC2492472 18663219

[26] Christov CP, Gardiner TJ, Szüts D, Krude T. Functional requirement of noncoding Y RNAs for human chromosomal DNA replication. Mol Cell Biol. 2006;26:6993-7004. 10.1128/MCB.01060-06 PMC1592862 16943439

[27] Dvinge H, Kim E, Abdel-Wahab O, Bradley RK. RNA splicing factors as oncoproteins and tumour suppressors. Nat Rev Cancer. 2016;16:413-430. 10.1038/nrc.2016.51 PMC5094465 27282250

[28] Hejret V, Varadarajan NM, Klimentova E, et al. Analysis of chimeric reads characterises the diverse targetome of AGO2-mediated regulation. Sci Rep. 2023;13:22895. 10.1038/s41598-023-49757-z PMC10739727 38129478

[29] Lambert M, Benmoussa A, Provost P. Small non-coding RNAs derived from eukaryotic ribosomal RNA. Noncoding RNA. 2019;5:16. 10.3390/ncrna5010016 PMC6468398 30720712

[30] Gaviraghi M, Vivori C, Tonon G. How cancer exploits ribosomal RNA biogenesis: a journey beyond the boundaries of rRNA transcription. Cells. 2019;8:1098. 10.3390/cells8091098 PMC6769540 31533350

[31] Diamantopoulos MA, Georgoulia KK, Levis P, et al. 28S rRNA-derived fragments represent an independent molecular predictor of short-term relapse in prostate cancer. Int J Mol Sci. 2023;25:239. 10.3390/ijms25010239 PMC10779029 38203408

[32] Fiskaa T, Knutsen E, Nikolaisen MA, et al. Distinct small RNA signatures in extracellular vesicles derived from breast cancer cell lines. PLoS One. 2016;11:e0161824. 10.1371/journal.pone.0161824 PMC5006963 27579604

[33] Fuchs RT, Sun Z, Zhuang F, Robb GB. Bias in ligation-based small RNA sequencing library construction is determined by adaptor and RNA structure. PLoS One. 2015;10:e0126049. 10.1371/journal.pone.0126049 PMC4420488 25942392

[34] Maguire S, Lohman GJS, Guan S. A low-bias and sensitive small RNA library preparation method using randomized splint ligation. Nucleic Acids Res. 2020;48:e80. 10.1093/nar/gkaa480 PMC7641310 32496547

[35] Vermeulen RJ, Andersson V, Banken J, et al. Limited generalizability and high risk of bias in multivariable models predicting conversion risk from mild cognitive impairment to dementia: a systematic review. Alzheimers Dement. 2025;21:e70069. 10.1002/alz.70069 PMC11972987 40189799

[36] Eminaga S, Christodoulou DC, Vigneault F, Church GM, Seidman JG. Quantification of microRNA expression with next-generation sequencing. Curr Protoc Mol Biol. 2013; 103:4.17.1-4.17.14. 10.1002/0471142727.mb0417s103 PMC4138881 23821442

[37] Martin M . Cutadapt removes adapter sequences from high-throughput sequencing reads. *EMBnet.journal.* 2011;17:10–12. 10.14806/ej.17.1.200

[38] Kozomara A, Birgaoanu M, Griffiths-Jones S. miRBase: from microRNA sequences to function. Nucleic Acids Res. 2019;47:D155-D162. 10.1093/nar/gky1141 PMC6323917 30423142

[39] Pantano L, Estivill X, Martí E. SeqBuster, a bioinformatic tool for the processing and analysis of small RNAs datasets, reveals ubiquitous miRNA modifications in human embryonic cells. Nucleic Acids Res. 2010;38:e34. 10.1093/nar/gkp1127 PMC2836562 20008100

[40] Wang C, Wallerman O, Arendt ML, et al. A novel canine reference genome resolves genomic architecture and uncovers transcript complexity. Commun Biol. 2021;4:185. 10.1038/s42003-021-01698-x PMC7875987 33568770

[41] Friedländer MR, Mackowiak SD, Li N, Chen W, Rajewsky N. miRDeep2 accurately identifies known and hundreds of novel microRNA genes in seven animal clades. Nucleic Acids Res. 2012;40:37-52. 10.1093/nar/gkr688 PMC3245920 21911355

[42] Li W, Godzik A. Cd-hit: a fast program for clustering and comparing large sets of protein or nucleotide sequences. Bioinformatics. 2006;22:1658-1659. 10.1093/bioinformatics/btl158 16731699

