# Supplementary material for: Novel liquid biopsy based on comprehensive small RNA profiling for screening of cancer in dogs utilizing machine learning
Source: J Vet Intern Med. 2026 Oct 30;40(5):aalag270. doi: 10.1093/jvimsj/aalag270 (PMC13720721; doi:10.1093/jvimsj/aalag270)
Supplement: Supplementary file 1 [file supplementary_material_aalag270.zip › Supplementary_material_caption.docx]

Supplementary Figure 1. Principal Component Analysis of the Top 50 Features. Analysis was performed on the log_2_ RPM values of the top 50 most highly expressed miRNAs and other small RNA species. No clear separation of clusters by age in months was observed for either miRNA or other small RNA species, indicating that the expression profiles were not strongly affected by age.

Supplementary Figure 2. Age-expression correlations of model features in positive and negative cohorts. Correlation coefficients between age and the expression level of each selected feature were calculated separately in the positive (cancer) and negative (healthy controls and non-malignant diseases) cohorts. Boxplots represent the distribution of correlation coefficients across all features.

Supplementary Figure 3. Comparison of Highly Expressed Features. A total of 88 miRNAs and 39 other small RNA species exhibited a read count of ≥20 in >90% of all samples. The expression of other small RNA species was also stable.

Supplementary Figure 4. Comparison of Sensitivity Distributions at a Fixed Specificity of .90. Figure shows the distributions of overall sensitivity and subgroup sensitivity for major cancer categories (mast cell tumor, malignant melanoma, and lymphoma) for models constructed using the miRNA-only and combined datasets. For lymphoma, sensitivity is shown from an additional validation analysis using a separate validation cohort. Methods using the same feature selection approach and algorithm are connected by solid lines. Overall sensitivity was significantly higher with the combined dataset than with the miRNA-only dataset (median: .791 [95% CI, .756–.844] vs. .676 [95% CI, .635–.718]; Wilcoxon signed-rank test, *p* = .003), and this improvement was also observed across the evaluated major cancer categories (**p* < .05, ***p* < .01).
