# Supplementary material for: Novel liquid biopsy based on comprehensive small RNA profiling for screening of cancer in dogs utilizing machine learning
Source: J Vet Intern Med. 2026 Oct 30;40(5):aalag270. doi: 10.1093/jvimsj/aalag270 (PMC13720721; doi:10.1093/jvimsj/aalag270)
Supplement: Supplementary file 1 [file supplementary_material_aalag270.zip › sup tabs.docx]

**SUPPLEMENTARY TABLES**

Supplementary Table 1. Number of Selected Features and AUC Values. Table shows the median AUC values from five-fold cross-validation and the number of miRNAs and other small RNA species used in each of the 12 classification models. In all models constructed using the combined dataset, other small RNA species accounted for 27–52% of features.

| **Model ID** | **Feature selection** | **Modeling algorithm** | **Number of features** | **AUC [95% CI]** |
| --- | --- | --- | --- | --- |
| M01M | Reads & Lasso,  50 features | ENET | 52 (miRNA: 52) | .898 [.842–.946] |
| M01C |  |  | 51 (miRNA: 29, other small RNA species: 22) | .931 [.879–.973] |
| M02M | Reads & Lasso,  50 features | SVM | 50 (miRNA: 50) | .921 [.872–.963] |
| M02C |  |  | 51 (miRNA: 29, other small RNA species: 22) | .947 [.909–.979] |
| M03M | Reads & Lasso,  50 features | XGB | 50 (miRNA: 50) | .880 [.824–.929] |
| M03C |  |  | 52 (miRNA: 33, other small RNA species: 19) | .912 [.840–.969] |
| M04M | Reads & Lasso,  30 features | ENET | 32 (miRNA: 32) | .883 [.821–.934] |
| M04C |  |  | 31 (miRNA: 15, other small RNA species: 16) | .960 [.927–.986] |
| M05M | Reads & Lasso,  30 features | SVM | 32 (miRNA: 32) | .891 [.829–.941] |
| M05C |  |  | 31 (miRNA: 15, other small RNA species: 16) | .955 [.917–.984] |
| M06M | Reads & Lasso,  30 features | XGB | 31 (miRNA: 31) | .846 [.751–.927] |
| M06C |  |  | 32 (miRNA: 19, other small RNA species: 13) | .909 [.840–.964] |
| M07M | Reads & F-score,  50 features | ENET | 50 (miRNA: 50) | .897 [.831–.952] |
| M07C |  |  | 50 (miRNA: 35, other small RNA species: 15) | .932 [.878–.976] |
| M08M | Reads & F-score,  50 features | SVM | 50 (miRNA: 50) | .920 [.869–.963] |
| M08C |  |  | 50 (miRNA: 34, other small RNA species: 16) | .940 [.896–.974] |
| M09M | Reads & F-score,  50 features | XGB | 50 (miRNA: 50) | .849 [.759–.925] |
| M09C |  |  | 50 (miRNA: 35, other small RNA species: 15) | .865 [.778–.936] |
| M10M | Reads & F-score,  30 features | ENET | 30 (miRNA: 30) | .871 [.792–.938] |
| M10C |  |  | 30 (miRNA: 20, other small RNA species: 10) | .921 [.869–.963] |
| M11M | Reads & F-score,  30 features | SVM | 30 (miRNA: 30) | .878 [.817–.930] |
| M11C |  |  | 30 (miRNA: 20, other small RNA species: 10) | .914 [.860–.960] |
| M12M | Reads & F-score,  30 features | XGB | 30 (miRNA: 30) | .845 [.756–.923] |
| M12C |  |  | 30 (miRNA: 22, other small RNA species: 8) | .866 [.776–.941] |

Supplementary Table 2. Overall and Cancer Type–Specific Sensitivity Analyses at a Fixed Specificity of .90. To assess overall and cancer type–specific sensitivity, analyses were performed for the full cohort as well as for melanoma, mast cell tumor, and lymphoma. The melanoma and mast cell tumor subgroups consisted of oral melanoma and cutaneous mast cell tumor cases, respectively, which are recognized clinical presentations of these cancer types.^S9,S10^ For the overall cohort and these two subgroups, sensitivity was estimated using five-fold cross-validation. Because the model-development cohort included only two lymphoma cases, lymphoma was evaluated separately using the model developed in the main analysis and a temporally separate validation cohort. These lymphoma cases were collected between November 2024 and June 2025 at the same institution using the same protocols as those described in the main text. In all analyses, sensitivity was evaluated at a specificity of .90.

| **Model ID** | **Overall**  **(n = 85)** | **Mast cell tumor**  **(n = 12)** | **Melanoma**  **(n = 17)** | **Lymphoma**  **(n = 12)** | **SD**  **(across subgroup)** |
| --- | --- | --- | --- | --- | --- |
| M01M | .694 | .792 | .656 | .750 | .069 |
| M01C | .835 | .792 | .875 | .833 | .042 |
| M02M | .724 | .792 | .656 | .833 | .093 |
| M02C | .865 | .833 | .875 | .875 | .024 |
| M03M | .712 | .792 | .656 | .708 | .068 |
| M03C | .794 | .792 | .781 | .917 | .075 |
| M04M | .647 | .708 | .625 | .667 | .042 |
| M04C | .729 | .667 | .750 | .833 | .083 |
| M05M | .724 | .708 | .719 | .750 | .022 |
| M05C | .835 | .833 | 1.000 | .875 | .087 |
| M06M | .753 | .750 | .781 | .833 | .042 |
| M06C | .865 | .875 | .938 | .917 | .032 |
| M07M | .694 | .750 | .688 | .708 | .032 |
| M07C | .771 | .792 | .750 | .917 | .087 |
| M08M | .659 | .750 | .594 | .708 | .081 |
| M08C | .724 | .750 | .625 | .875 | .125 |
| M09M | .635 | .750 | .563 | .750 | .108 |
| M09C | .788 | .750 | .875 | .917 | .087 |
| M10M | .635 | .750 | .625 | .792 | .087 |
| M10C | .853 | .917 | .875 | .917 | .024 |
| M11M | .576 | .583 | .500 | .708 | .105 |
| M11C | .747 | .875 | .688 | .917 | .122 |
| M12M | .559 | .667 | .531 | .667 | .078 |
| M12C | .765 | .750 | .781 | .917 | .089 |

Supplementary Table 3. Relationship Between Prediction Score and Age (in months), Sex, and Dog Breed. No meaningful correlations were observed in any of the 24 models (r = .04–.29), and there were no significant differences between sexes (Wilcoxon rank-sum test, minimum *p* = .11). For breeds with at least 10 dogs, a significant difference was observed only in model M01C among the 24 models (Kruskal-Wallis test, *p* = .03); no significant differences were observed in the other 23 models (minimum *p* = .06). Additional analysis (Dunn’s test) revealed a significant difference between Miniature Dachshunds and other breeds (Chihuahua vs. Miniature Dachshund, *p* = .02; Chihuahua vs. Toy Poodle, *p* = .84; Miniature Dachshund vs. Toy Poodle, *p* = .02). Subsequently, to determine whether the Miniature Dachshund breed had a greater impact on prediction score than cancer status itself in model M01C, we examined the distribution of scores between the positive and negative groups within this breed. Discrimination between the cancer and non-cancer groups remained significant among Miniature Dachshunds (Wilcoxon rank-sum test, *p*<.001), even though breed was identified as a possible confounder.

| **Model ID** | **Class** | **Age (r)** | **Sex (*p*)** | **Breed (*p*)** |
| --- | --- | --- | --- | --- |
| M01M | Positive | .07 | .99 | .23 |
|  | Negative | .18 | .65 | .56 |
| M01C | Positive | .16 | .54 | .83 |
|  | Negative | .11 | .15 | .03 |
| M02M | Positive | .10 | .60 | .14 |
|  | Negative | .22 | .82 | .82 |
| M02C | Positive | .16 | .93 | .62 |
|  | Negative | .18 | .93 | .60 |
| M03M | Positive | .15 | .54 | .20 |
|  | Negative | .21 | .80 | .27 |
| M03C | Positive | .12 | .27 | .88 |
|  | Negative | .14 | .33 | .81 |
| M04M | Positive | .04 | .85 | .13 |
|  | Negative | .16 | .89 | .57 |
| M04C | Positive | .17 | .28 | .92 |
|  | Negative | .05 | .17 | .11 |
| M05M | Positive | .04 | .81 | .46 |
|  | Negative | .19 | .13 | .58 |
| M05C | Positive | .18 | .69 | .64 |
|  | Negative | .13 | .81 | .68 |
| M06M | Positive | .04 | .92 | .31 |
|  | Negative | .17 | .89 | .55 |
| M06C | Positive | .15 | .32 | .33 |
|  | Negative | .19 | .26 | .57 |
| M07M | Positive | .12 | .67 | .06 |
|  | Negative | .23 | .46 | .48 |
| M07C | Positive | .14 | .51 | .72 |
|  | Negative | .29 | .64 | .96 |
| M08M | Positive | .12 | .66 | .06 |
|  | Negative | .28 | .70 | .84 |
| M08C | Positive | .11 | .62 | .68 |
|  | Negative | .26 | .72 | .99 |
| M09M | Positive | .13 | .95 | .08 |
|  | Negative | .16 | .11 | .92 |
| M09C | Positive | .08 | .66 | .85 |
|  | Negative | .18 | .72 | .99 |
| M10M | Positive | .13 | .94 | .14 |
|  | Negative | .22 | .43 | .47 |
| M10C | Positive | .15 | .53 | .94 |
|  | Negative | .20 | .39 | .18 |
| M11M | Positive | .15 | .91 | .07 |
|  | Negative | .28 | .95 | .82 |
| M11C | Positive | .16 | .69 | .57 |
|  | Negative | .21 | .22 | .86 |
| M12M | Positive | .12 | .98 | .07 |
|  | Negative | .23 | .89 | .87 |
| M12C | Positive | .10 | .88 | .79 |
|  | Negative | .21 | .95 | .63 |
